# Novel Spectrophotometric Method for Robust Detection of Trace Copper and Cobalt in High-Concentration Zinc Solution

**DOI:** 10.3390/molecules29235765

**Published:** 2024-12-06

**Authors:** Fengbo Zhou, Bo Wu, Jianhua Zhou

**Affiliations:** Hunan Province Key Laboratory of Southwest, Hunan Academician Workstation, School of Information Science and Engineering, Shaoyang University, Shaoyang 422000, China; bwu4580@126.com (B.W.); fb.john@hnsyu.edu.cn (J.Z.)

**Keywords:** zinc hydrometallurgy, continuous wavelet transform, derivative spectrophotometry, multi-objective optimization, ultraviolet–visible spectroscopy

## Abstract

In the purification process of zinc hydrometallurgy, the spectra of copper and cobalt seriously overlap in the whole band and are interfered with by the spectra of zinc and nickel, which seriously affects the detection results of copper and cobalt in zinc solutions. Aiming to address the problems of low resolution, serious overlap, and narrow characteristic wavelengths, a novel spectrophotometric method for the robust detection of trace copper and cobalt is proposed. First, the Haar, Db4, Coif3, and Sym3 wavelets are used to carry out the second-order continuous wavelet transform on the spectral signals of copper and cobalt, which improves the resolution of copper and cobalt and eliminates the background interference caused by matrix zinc signals and reagents. Then, the information ratio and separation degree are defined as optimization indexes, a multi-objective optimization model is established with the wavelet decomposition scale as a variable, and the non-inferior solution of multi-objective optimization is solved by the state transition algorithm. Finally, the optimal second-derivative spectra combined with the fine zero-crossing technique are used to establish calibration curves at zero-crossing points for the simultaneous detection of copper and cobalt. The experimental results show that the detection performance of the proposed method is far superior to the partial least squares and Kalman filtering methods. The RMSEPs of copper and cobalt are 0.098 and 0.063, the correlation coefficients are 0.9953 and 0.9971, and the average relative errors of copper and cobalt are 3.77% and 2.85%, making this method suitable for the simultaneous detection of trace copper and cobalt in high-concentration zinc solutions.

## 1. Introduction

The zinc hydrometallurgy process mainly includes roasting, leaching, purification, and electrolysis. Firstly, zinc concentrate is roasted by boiling it to obtain zinc (Zn) oxide and other metal oxides, such as cobalt (Co) oxide, copper (Cu) oxide, and nickel (Ni) oxide [1,2,3,4]. Secondly, diluted sulfuric acid is used to add acid to the roasted product for dissolution, and the solid residue of the acid-insoluble nonmetallic oxide is removed through solid–liquid separation to obtain a neutral supernatant [5,6,7,8]. Then, based on the zinc powder replacement method, the neutral supernatant is purified to remove impurities such as copper, cobalt, and nickel [9,10,11]. Finally, the purified zinc sulfate solution is electrodeposited to obtain high-quality solid zinc [12,13,14]. In the electrolytic process of zinc hydrometallurgy, if the concentration of impure ions such as copper, cobalt, and nickel in zinc sulfate solution exceeds the standard, the phenomenon of “plate burning” occurs, and the current efficiency is significantly reduced by mild plate burning, while serious plate burning leads to the shutdown and maintenance of the electrolytic cell [15,16,17,18,19,20]. Therefore, the purification process is the key process of zinc hydrometallurgy. It is a prerequisite to ensure the normal operation of the zinc hydrometallurgy process by accurately detecting the concentration of impurity ions in the electrolyte and then removing the impurity ions based on the zinc powder replacement method. However, at present, enterprises still use the manual offline analysis of the concentration of impurity metal ions in the electrolyte, which is cumbersome and time-consuming and comes with delays in obtaining feedback information; it is also impossible to optimize and adjust the process parameters of the purification process in real time with this type of analysis [21,22,23,24]. Therefore, it is urgent to realize the online detection of the concentration of impurity metal ions in a zinc electrolyte, as this would provide a guarantee for the real-time control and optimal operation of production processes.

Ultraviolet–visible spectrometry has been widely used in low-concentration detection because of its high detection accuracy, the short period required, the fact that it is a simple analysis method, its strong on-line detection ability and its real-time feedback ability [25,26,27,28]. In order to realize the simultaneous detection of impurity ions of copper and cobalt in high-zinc solutions, it is necessary to choose the optimal chromogenic reaction system to allow the polymetallic ions to be detected from different complexes, so that the spectral characteristics between ions will be more obvious and can appear at a greater resolution. The chromogenic reaction system mainly includes a chromogenic agent and buffer [29,30]. After a lot of experiments, our team chose Nitroso R salt as the chromogenic reagent and acetic acid–sodium acetate (pH = 5.5) as the buffer reagent. However, in the purification process of zinc hydrometallurgy, there are some difficulties in detecting the concentration of polymetallic impurity ions in zinc sulfate solution by ultraviolet–visible spectrometry: (1) the concentration ratio of matrix zinc to impurity metal ions is as high as 10^5^, and the spectral signal is seriously masked; (2) the chemical characteristics of polymetallic ions are similar, the effective band is narrow, and the spectral characteristic information is concentrated, which makes the spectral signals overlap and interfere seriously; (3) with the increase in particle density in solution, the absorption rate and transmittance of incident light are affected, which leads to serious deviation in the relationship between the absorbance and concentration of the mixture by the Beer–Lambert law; (4) under the effects of instrument circuit noise, light source fluctuation, matrix zinc ion interference, and other factors, there are problems of range overflow and poor resolution, so it is difficult to obtain polymetallic spectral signals online [3,31,32,33]. These problems pose great challenges to the simultaneous determination of polymetallic impurity ions in high-zinc solutions.

In order to solve the problems of low sensitivity, serious spectral overlap, and narrow effective spectral bands of copper and cobalt in highly concentrated zinc solutions, a novel spectrophotometric method for the robust detection of trace copper and cobalt is proposed. First, Haar, Db4, Coif3, and Sym3 wavelets are used to carry out the second-order continuous wavelet transform on the spectral signals of copper and cobalt, improving the resolution of copper and cobalt and eliminating the background interference caused by matrix zinc signals and reagents. Then, the information ratio and separation degree are defined as optimization indexes, the multi-objective optimization model is established with the wavelet decomposition scale as a variable, and the non-inferior solution of multi-objective optimization is solved by the state transition algorithm. Finally, the optimal second-derivative spectra combined with the fine zero-crossing technique are used to establish calibration curves at zero-crossing points for the simultaneous detection of copper and cobalt [34,35,36,37,38,39]. The experimental results show that the detection performance of the proposed method is far superior to the partial least squares and Kalman filtering methods, and it is suitable for the simultaneous detection of copper and cobalt in high-concentration zinc solutions.

## 2. Theory

### 2.1. Derivative Spectrophotometry

In zinc sulfate solutions, the sensitivity of copper and cobalt is low, the effective band is narrow, and the spectral signal is completely covered by the zinc signal. Therefore, derivative spectrometry is used to solve the problem of complete coverage of the copper and cobalt spectral signals. According to the spectral characteristics of the mixed solution, when the wavelength exceeds 500 nm, the absorbance of zinc and nickel are almost constant and approach zero. Therefore, in the wavelength range of 500–600 nm, the absorbance of the mixed solution is given as

(1) $$A_{\lambda_{i}}=\varepsilon_{M,\lambda_{i}}C_{M}+\varepsilon_{N,\lambda_{i}}C_{N}{+A}_{d},$$

where $C_{M}$ and $C_{N}$ represent the concentrations of Cu and Co, and $\varepsilon_{M,\lambda_{i}}$ and $\varepsilon_{N,\lambda_{i}}$ indicate the absorptivity of Cu and Co at wavelength $\lambda_{i}$, respectively. $A_{\lambda_{i}}$ indicates the absorbance of the mixed solution, and $A_{d}$ stands for background interference (including the absorbance of zinc and nickel). The derivation of Equation (1) is as follows

(2) $$\frac{d(A_{\lambda_{i}})}{d\lambda }=\frac{d(\varepsilon_{M,\lambda_{i}}C_{M})}{d\lambda }+\frac{d(\varepsilon_{N,\lambda_{i}}C_{N})}{d\lambda }+\frac{d{(A}_{d})}{d\lambda }.$$

Since $A_{d}$ changes very slowly with the wavelength, it can be considered a constant. Equation (2) can be expressed as

(3) $$\frac{d(A_{\lambda_{i}})}{d\lambda }=\frac{d(\varepsilon_{M,\lambda_{i}}C_{M})}{d\lambda }+\frac{d(\varepsilon_{N,\lambda_{i}}C_{N})}{d\lambda }.$$

In Equation (3), the derivative spectra of the four mixtures of Zn, Cu, Co, and Ni depend entirely on the concentrations of Cu and Co and have nothing to do with the concentrations of Zn and Ni. Therefore, the derivative spectra obtained can completely eliminate the absorbance contributions of Zn and Ni. By using the zero-crossing technique, at the zero-crossing wavelength of Co, the derivative spectra of the mixture is only proportional to the concentration of Cu. Similarly, at the zero-crossing wavelength of Cu, the derivative spectra of the mixture is only proportional to the concentration of Co. Therefore, derivative spectrometry combined with the zero-crossing technique can obtain the calibration curves of Cu and Co and realize the simultaneous detection of Cu and Co in zinc solutions.

### 2.2. Multi-Objective Optimization Model

With derivative spectrophotometry, it is easy to amplify the noise signal, so this paper uses the approximate derivative method based on the continuous wavelet transform (CWT). Because the wavelet function and decomposition scale of the continuous wavelet transform affect the peak amplitude and peak position of spectral signals, in order to further improve the sensitivity and separation of the Cu and Co derivative spectral signals, a multi-objective optimization model is established with the wavelet decomposition scale as a variable and the information ratio and separation degree as optimization indexes. The information ratio is used to characterize the sensitivity and interference degree of the ion to be measured, and the separation degree is used to characterize the overlapping resolution between the metal ions to be measured.

When the absorbance of the ion to be measured at a certain wavelength is greater than that of other ions, the wavelength is called the effective wavelength point. The more effective the wavelength points, the higher the sensitivity of the ion to be measured. Therefore, the information ratio is defined as the ratio of the effective wavelength number to the total wavelength number, where the effective wavelength number is obtained by the absorbance ratio matrix of the spectral signal, and the formula is as follows:

(4) $$y^{{Abs\_ratio}_{k\times n}}=\left[\begin{array}{ccc} \frac{y^{{Abs}_{11}}}{\sum_{i=1}^{k}y^{{Abs}_{i1}}} & \cdots & \frac{y^{{Abs}_{1n}}}{\sum_{i=1}^{k}y^{{Abs}_{in}}}\\ \vdots & \ddots & \vdots \\ \frac{y^{{Abs}_{k1}}}{\sum_{i=1}^{k}y^{{Abs}_{i1}}} & \cdots & \frac{y^{{Abs}_{kn}}}{\sum_{i=1}^{k}y^{{Abs}_{in}}} \end{array}\right]$$

In Equation (4), the absorbance data are all from the mixed solution of Cu and Co at the same concentration in a high-concentration zinc solution. If the ratio of the absorbance of the ion to be measured to the total absorbance is greater than 0.5, the corresponding wavelength is the effective wavelength, so that the number of all effective wavelengths can be counted; then, the information ratio of the ion to be measured can be calculated.

In order to measure the overlapping resolution of spectral signals, the separation degree is used to evaluate the peak separation degree of Cu and Co. Separation degree is defined as the ratio of the peak spacing to the peak width sum of Cu and Co. The greater the separation degree, the smaller the interference between Cu and Co.

**Figure 1:**
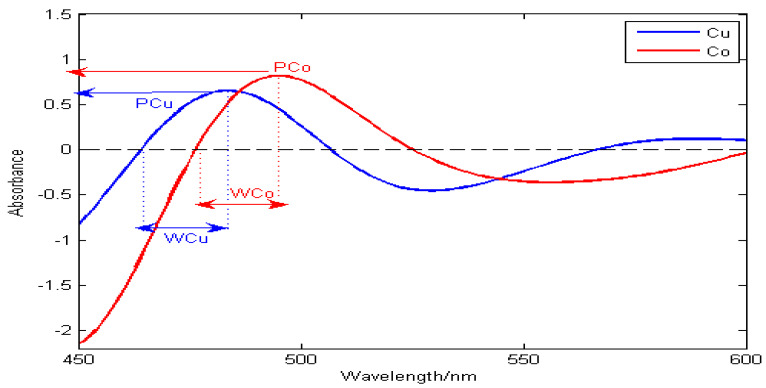
Schematic diagram of peak position resolution.

In Figure 1, $P_{Cu}$ and $P_{Co}$ represent the peak positions of Cu and Co, and $W_{Cu}$ and $W_{Co}$ represent the half-peak widths of Cu and Co, respectively. The separation degree RP is defined as

(5) $$RP=\frac{\left|P_{Cu}-P_{Co}\right|}{W_{Cu}+W_{Co}}.$$

The greater the value of RP, the higher the resolution of the spectral peaks. When 0≤RP<1, the spectral signals of Cu and Co overlap. When RP≥1, it shows that the spectral signals of Cu and Co are completely separated.

In the derivative spectral signal of Cu and Co, with the decomposition scale x as the decision variable and the information ratio F(x) and separation degree G(x) as the optimization objectives, the multi-objective optimization models are expressed by the following Formulas (6) and (7), respectively.

(6) $$\begin{array}{l} \max{\;J}_{1}(x)=F_{1}(x)\;,\\ \max{\;J}_{2}(x)=G(x)\;,\\ s.t.\;10\leq x\leq 100\;. \end{array}$$

(7) $$\begin{array}{l} \max{\;J}_{1}(x)=F_{2}(x)\;,\\ \max{\;J}_{2}(x)=G(x)\;,\\ s.t.\;10\leq x\leq 100\;. \end{array}$$

where $F_{1}(x)$ represents the information ratio of Cu, $F_{2}(x)$ indicates the information ratio of Co, and G(x) stands for the separation degree of Cu and Co. The state transition algorithm (STA) is a global optimization method which can quickly find the global optimal solution and avoid falling into local optimal solution. In this paper, the state transition algorithm (STA) is used to solve the multi-objective optimization model.

## 3. Results and Discussion

### 3.1. Spectral Characteristics

**Figure 2:**
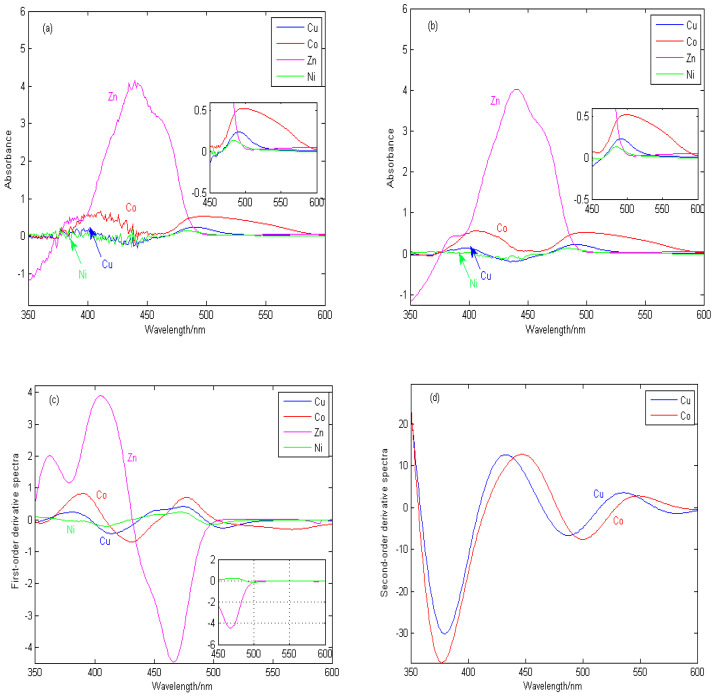
Experimental spectral signals and their derivative spectral signals. (**a**) The original spectral signals. (**b**) Spectral pretreatment signals. (**c**) First-order derivative spectra. (**d**) Second-order derivative spectra.

Figure 2 shows the original spectral signals, spectral pretreatment signals, first-derivative spectra, and second-order derivative spectra of Zn, Cu, Co, and Ni in zinc sulfate solution, in which the concentration of zinc is 20 g/L, and the concentrations of Cu, Co, and Ni are all 1.2 mg/L.

Figure 2a shows the experimental spectral signals of Zn, Cu, Co, and Ni. Because the concentration of Zn in zinc sulfate solution is much greater than those of Cu, Co, and Ni, the absorbance of Zn is much greater than that of Cu and Co at 350–500 nm, resulting in low sensitivity and poor linearity of Cu and Co. When the wavelength exceeds 500 nm, the spectral absorbance of Zn and Ni is close to zero and tends to be unchanged, but the spectral signals of Cu and Co overlap seriously, and especially, the copper spectral signals are seriously covered by the cobalt spectra, which leads to the low sensitivity of copper signals.

Figure 2b shows the spectral signal pretreated by the adaptive wavelet threshold method, which is used to eliminate random noise during instrument measurement and improve the accuracy and repeatability of detection. Figure 2c shows the first-derivative spectra by the continuous wavelet transform. The Haar wavelet is used to approximate the derivative of the original spectral signal. When the wavelength is greater than 500 nm, the derivative spectra of Zn and Ni are close to zero, thus shielding the interference from Zn and Ni. In addition, the overlapping spectral signals of Cu and Co begin to separate, especially the characteristic information of Cu begins to stand out. In some wavelength bands, the absorbance of Cu is greater than that of Co, which is no longer completely covered by the cobalt signal, but the sensitivity of copper in the whole band is still low. Figure 2d shows the second-order derivative spectra obtained by the continuous wavelet transform. The overlapping signals of Cu and Co are obviously separated. In the wavelength range of 520–550 nm, the spectral peak of copper is completely exposed and is no longer covered by the cobalt signal, and the sensitivity is significantly improved. Therefore, the second-order derivative spectra can separate the overlapping signals of Cu and Co, reduce mutual interference, highlight the spectral peak information significantly, and especially improve the sensitivity of copper.

### 3.2. Second-Order Derivative Spectra of Copper and Cobalt

**Figure 3:**
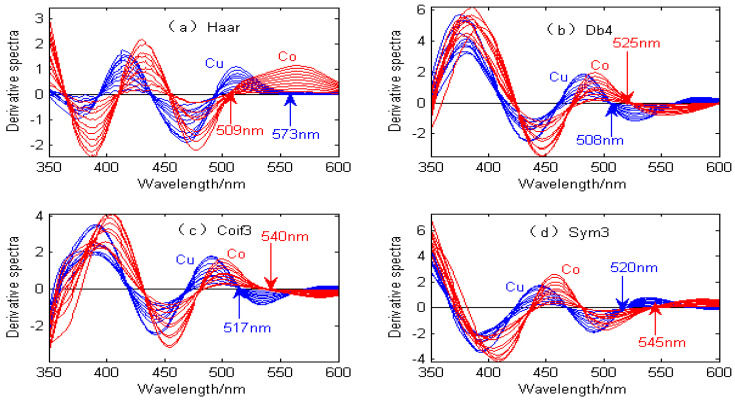
Derivative spectral signal by continuous wavelet transform with different wavelet functions.

The first-order continuous wavelet transform uses the Haar wavelet to approximate the derivative, which is mainly used to shield the influence of Zn and Ni and eliminate the interference from the reagent background. The spectral signal of the second-order continuous wavelet transform is used to separate the overlapping signals of Cu and Co to the greatest extent and reduce the interference between them, thus significantly improving the sensitivity and linearity of Cu and Co. The difficulty of the second-order continuous wavelet transform lies in the choice of the wavelet function and the decomposition scale. For copper and cobalt spectral signals, when the wavelet decomposition scale is fixed at 20, the continuous wavelet transform with different wavelet functions produces different results, as shown in Figure 3.

Figure 3 shows the derivative spectral signals of Cu and Co by the continuous wavelet transform with different wavelet functions. Figure 3a shows that when the Haar wavelet is used for the continuous wavelet transform, the derivative spectral signals of Cu and Co have characteristic peaks at 509 nm and 573 nm, respectively. Figure 3b shows that when the Db4 wavelet is used for the continuous wavelet transform, the derivative spectral signals of Cu and Co have characteristic peaks at 525 nm and 508 nm, respectively. Figure 3c shows that when the Coif3 wavelet is used for the continuous wavelet transform, the derivative spectral signals of Cu and Co have characteristic peaks at 540 nm and 517 nm, respectively. Figure 3d shows that when the Sym3 wavelet is used for the continuous wavelet transform, the derivative spectral signals of Cu and Co have characteristic peaks at 545 nm and 520 nm, respectively. As can be seen from Figure 3, the derivative spectral signals obtained by the continuous wavelet transform with different wavelet functions are completely different, and the biggest difference lies in the amplitude and position of the characteristic peaks of the spectral signals. Therefore, in order to improve the overlapping peak separation of Cu and Co, it is necessary to choose the optimal wavelet function.

### 3.3. Optimization Index

In order to solve the problem of the serious overlap of Cu and Co and the low sensitivity of the copper ion, the overlapping peak separation method based on derivative spectrometry is used. In order to further improve the sensitivity and resolution of the derivative spectral signals of Cu and Co, a multi-objective optimization model was established with the information ratio and separation degree as optimization indexes and the wavelet decomposition scale as the variable. Among them, the information ratio is used to characterize the sensitivity and interference degree of the ions to be measured, and the separation degree is used to characterize the overlapping resolution between the metal ions to be measured.

**Figure 4:**
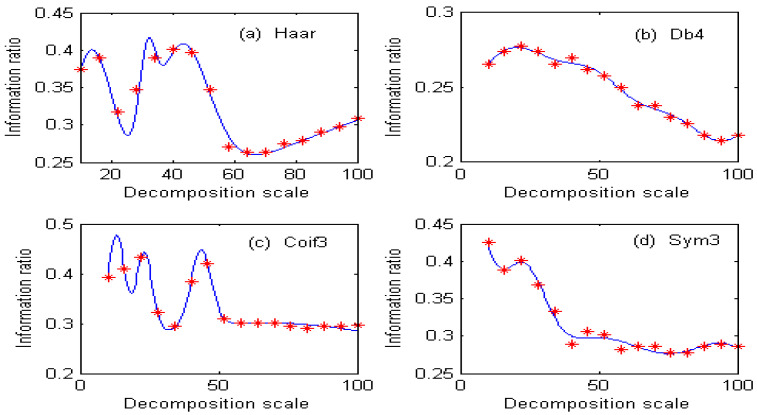
Fitting function of copper information ratio under different wavelet bases.

**Figure 5:**
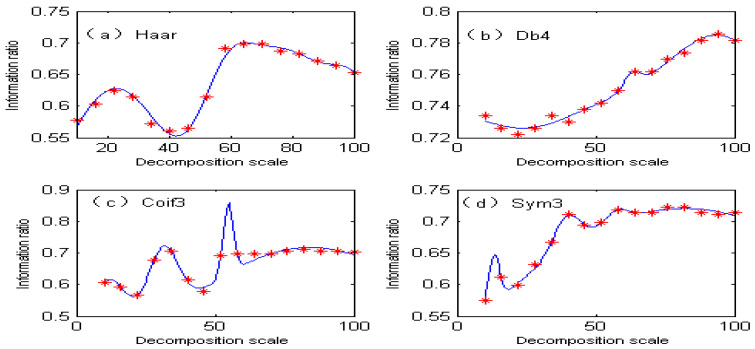
Fitting function of cobalt information ratio under different wavelet bases.

Under different decomposition scales, the separation degree of overlapping peaks of spectral signals obtained by the second-order continuous wavelet transform is different, so the information ratio of ions to be measured is also different. If the decomposition scale is represented by x, the information ratio of copper is represented by F_1_(x), the information ratio of cobalt is represented by F_2_(x), and the spectral peak separation of Cu and Co is represented by G(x). Commonly used wavelets are Haar, Db4, Coif3, and Sym3. The fitting function of the information ratio for copper under different wavelet bases is shown in Figure 4, and the fitting accuracy is shown in Table 1. The fitting function of the information ratio for cobalt under different wavelet bases is shown in Figure 5, and the fitting accuracy is shown in Table 2.

As can be seen from Table 1, the fitting accuracy of the copper information ratio is good, reaching above 0.99 under different wavelet functions, and the root mean square error (RMSE) is very small. As can be seen from Figure 4, the distribution of the copper information ratio is completely different under different wavelet functions. Figure 4a uses the Haar wavelet function. With the increase in the decomposition scale, the information ratio of copper changes very sharply, and the information ratio value is higher when the decomposition scale is 30–60. Figure 4b shows the use of the Db4 wavelet function. The overall information ratio of copper shows a decreasing trend, and the maximum value is only 0.28. Figure 4c shows the use of the Coif3 wavelet function. The information ratio fluctuates greatly, and the information ratio changes slowly when the decomposition scale is greater than 50. Figure 4d shows the use of the Sym3 wavelet function. The information ratio decreases rapidly. Because of the low sensitivity of copper, it is seriously masked by cobalt at the same concentration, so the information ratio of copper after the second-order wavelet transform is small.

From Table 2, it can be seen that the fitting accuracy of the cobalt information ratio is good and is as high as 0.99 or above under different wavelet functions. Figure 5 shows the fitting function of the information ratio for cobalt under different wavelet functions. As can be seen from Figure 5, under different wavelet functions, the information ratio of cobalt generally has a higher value, because the sensitivity of cobalt is better than that of copper, so there are more effective wavelengths.

**Figure 6:**
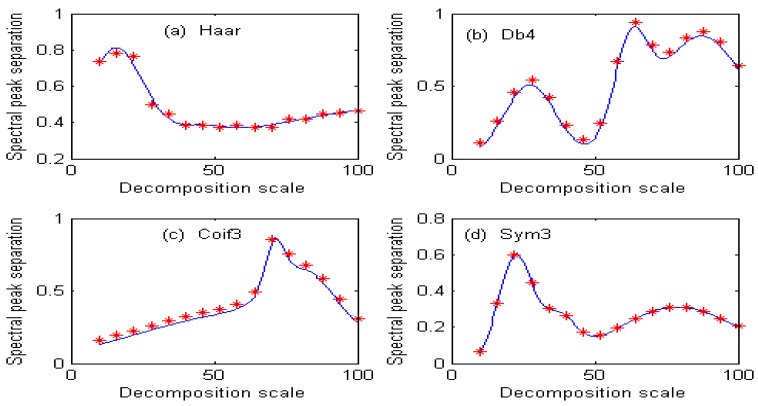
Fitting function of separation degree of copper and cobalt under different wavelet functions.

Figure 6 shows the fitting function of the separation degree of Cu and Co under different wavelet functions. From Table 3, it can be seen that the fitting accuracy of the separation degree G(x) of Cu and Co are good.

### 3.4. Non-Inferior Solution of Multi-Objective Optimization

**Figure 7:**
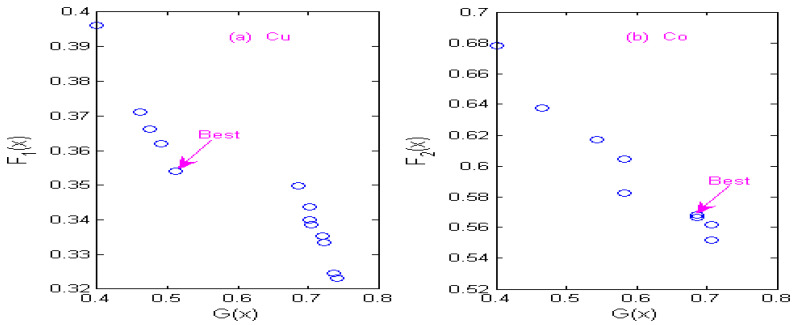
Non-inferior solution sets of copper and cobalt under Haar wavelet. (**a**) Non-inferior solution sets of copper. (**b**) Non-inferior solution sets of cobalt.

**Figure 8:**
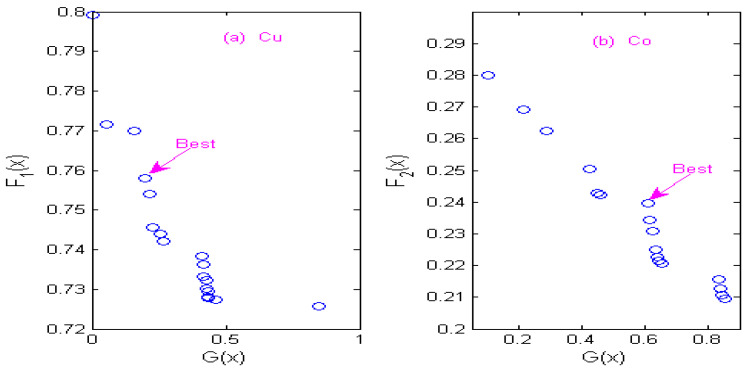
Non-inferior solution sets of copper and cobalt under Db4 wavelet. (**a**) Non-inferior solution sets of copper. (**b**) Non-inferior solution sets of cobalt.

**Figure 9:**
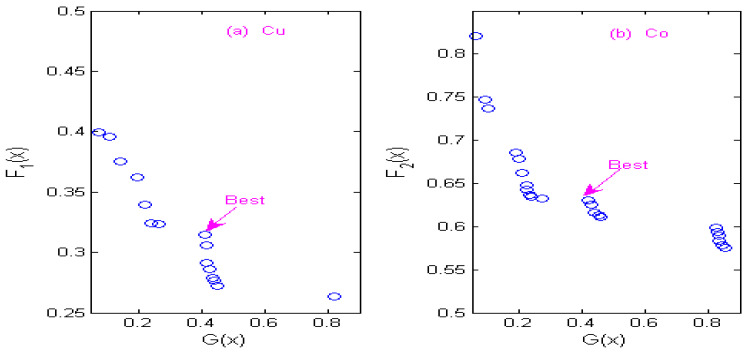
Non-inferior solution sets of copper and cobalt under Coif3 wavelet. (**a**) Non-inferior solution sets of copper. (**b**) Non-inferior solution sets of cobalt.

**Figure 10:**
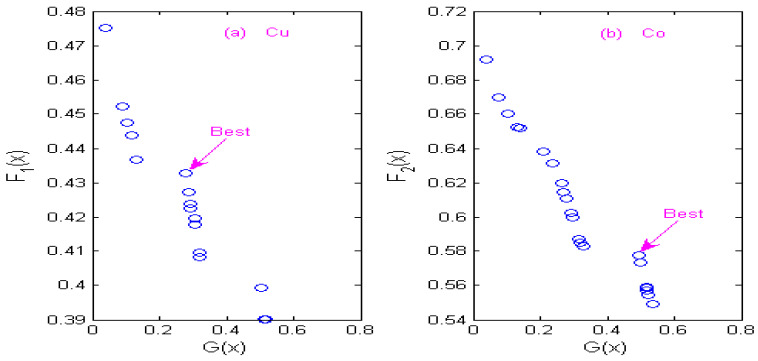
Non-inferior solution sets of copper and cobalt under Sym3 wavelet. (**a**) Non-inferior solution sets of copper. (**b**) Non-inferior solution sets of cobalt.

The state transition algorithm (STA) is used to solve two constrained multi-objective optimization models of Cu and Co, and non-inferior solution sets of Cu and Co under different wavelets are obtained according to the STA algorithm. Figure 7 shows the non-inferior solution sets of Cu and Co by using the Haar wavelet. Figure 8 shows the non-inferior solution sets of Cu and Co by using the Db4 wavelet; Figure 9 shows the non-inferior solution sets of Cu and Co by using the Coif3 wavelet; Figure 10 shows the non-inferior solution sets of Cu and Co by using the Sym3 wavelet. Because the spectral signals of Cu and Co overlap seriously, the sensitivity of Co is higher than that of Cu at the same concentration, so it is more inclined towards the non-inferior solution with large spectral peak separation in the non-inferior solution sets. Cu has low sensitivity, so it is more inclined towards the non-inferior solution with a large information ratio. Therefore, according to the spectral characteristics of Cu and Co, when the Haar wavelet is used for the continuous wavelet transform, the optimal decomposition scale of copper is x = 30, and that of cobalt is x = 67; When the Db4 wavelet is used for the continuous wavelet transform, the optimal decomposition scale of copper is x = 55, and that of cobalt is x = 42. When the Coif3 wavelet is used for the continuous wavelet transform, the optimal decomposition scale of copper is x = 60, and that of cobalt is x = 53. When using the Sym3 wavelet for the continuous wavelet transform, the optimal decomposition scale of copper ion is x = 47, and that of cobalt ion is x = 69.

### 3.5. Derivative Spectral Correction Curve

**Figure 11:**
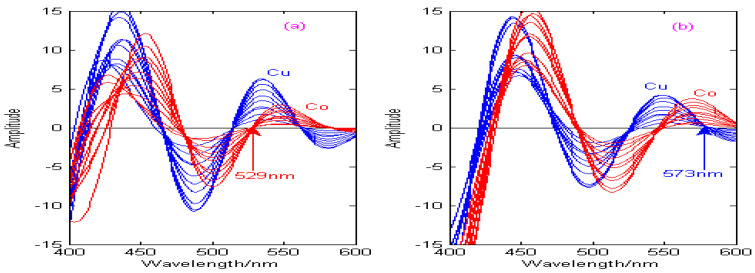
Calibration curves of copper and cobalt under the Haar wavelet. (**a**) The decomposition scale x for copper is 30. (**b**) The decomposition scale x for cobalt is 67.

**Figure 12:**
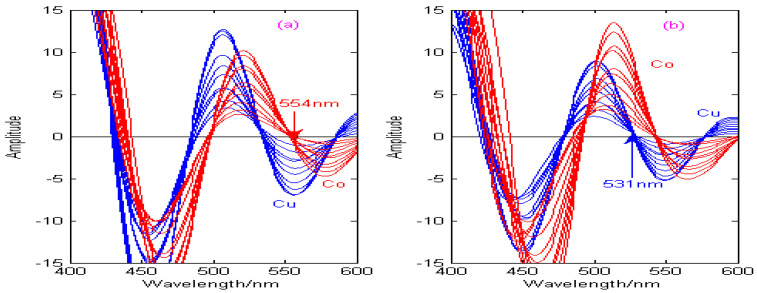
Calibration curves of copper and cobalt under the Db4 wavelet. (**a**) The decomposition scale x for copper is 55. (**b**) The decomposition scale x for cobalt is 42.

**Figure 13:**
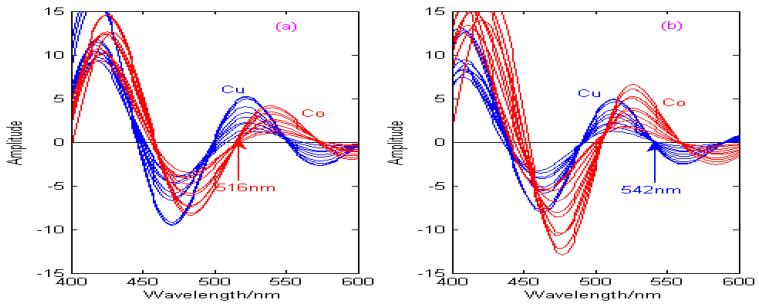
Calibration curves of copper and cobalt under the Coif3 wavelet. (**a**) The decomposition scale x for copper is 60. (**b**) The decomposition scale x for cobalt is 53.

**Figure 14:**
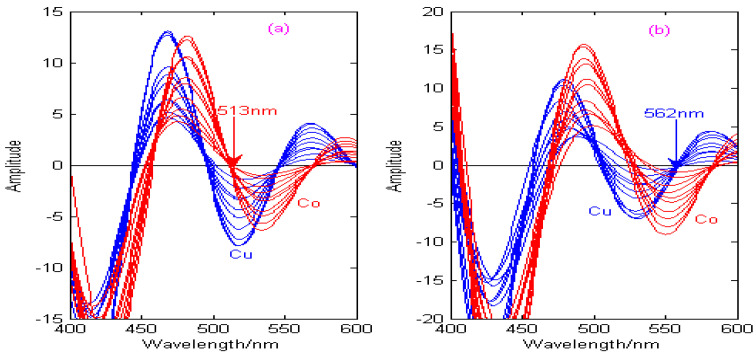
Calibration curves of copper and cobalt under the Sym3 wavelet. (**a**) The decomposition scale x for copper is 47. (**b**) The decomposition scale x for cobalt is 69.

The Haar, Db4, Coif3, and Sym3 wavelets are used to carry out the second-order continuous wavelet transform on the spectral signals of Cu and Co, and the state transition multi-objective optimization algorithm is applied to solve the optimal decomposition scale of Cu and Co under different wavelet bases, which can effectively improve the resolution of the copper and cobalt overlapping signals. In this paper, the second-order continuous wavelet transform combined with the zero-crossing technique can be used to establish the calibration curves of copper and cobalt, as shown in Figure 11, Figure 12, Figure 13 and Figure 14.

As can be seen from Figure 11, Figure 12, Figure 13 and Figure 14, the spectral signals of Cu and Co are processed by the Haar, Db4, Coif3, and Sym3 wavelet functions, and the concentrations of Cu and Co can be determined simultaneously by combining the zero-crossing technology. Figure 11 shows the second-derivative spectra of Cu and Co, which are obtained by the Haar wavelet. Figure 11a shows that the optimal decomposition scale x is 30, which is beneficial for establishing the calibration curve of Cu at the zero-crossing wavelength (529 nm) of Co. Figure 11b shows that the optimal decomposition scale x is 67, which is beneficial for establishing the calibration curve of Co at the zero-crossing wavelength (573 nm) of Cu. Figure 12 shows the second-derivative spectra of Cu and Co, which are obtained by the Db4 wavelet. Figure 12a shows that the optimal decomposition scale x is 55, which is beneficial for establishing the calibration curve of Cu at the zero-crossing wavelength (554 nm) of Co. Figure 12b shows that the optimal decomposition scale x is 42, which is beneficial for establishing the calibration curve of Co at the zero-crossing wavelength (531 nm) of Cu. Figure 13 shows the second-derivative spectra of Cu and Co, which are obtained by the Coif3 wavelet. Figure 13a shows that the optimal decomposition scale x is 60, which is beneficial for establishing the calibration curve of Cu at the zero-crossing wavelength (516 nm) of Co. Figure 13b shows that the optimal decomposition scale x is 53, which is beneficial for establishing the calibration curve of Co at the zero-crossing wavelength (542 nm) of Cu. Figure 14 shows the second-derivative spectra of Cu and Co, which are obtained by the Sym3 wavelet. Figure 14a shows that the optimal decomposition scale x is 47, which is beneficial for establishing the calibration curve of Cu at the zero-crossing wavelength (513 nm) of Co. Figure 14b shows that the optimal decomposition scale x is 69, which is beneficial for establishing the calibration curve of Co at the zero-crossing wavelength (562 nm) of Cu. Therefore, the calibration curves of Cu and Co can be obtained by derivative spectrometry combined with zero-crossing technology, and the simultaneous detection of Cu and Co in zinc solution can be realized. The correction equations of Cu and Co under different wavelet functions are shown in Table 4.

As can be seen from Table 4, compared with the Db4, Coif3, and Sym3 wavelets, the copper ion has the highest accuracy in the calibration curve established by the Haar wavelet, with the detection limit (LOD) of 0.0829, the quantitative detection limit (LOQ) of 0.2733, and the correlation coefficient (R^2^) of 0.9952, which indicates that the copper ion has a lower detection limit and better linearity when using the Haar wavelet. Compared with the Haar, Db4, and Sym3 wavelets, the calibration curve of the cobalt ion by using the Coif3 wavelet has the highest accuracy, with a detection limit (LOD) of 0.0788, a quantitative detection limit (LOQ) of 0.2575, and a correlation coefficient (R^2^) of 0.9963, which indicates that the cobalt ion has a lower detection limit and better linearity when using the Coif3 wavelet. Therefore, the Haar wavelet and Coif3 wavelet are selected to detect the concentrations of Cu and Co by derivative spectrometry, respectively.

### 3.6. Simultaneous Determination of Copper and Cobalt

In order to verify the effectiveness of the proposed spectrophotometric method, partial least squares (PLS) and Kalman filter spectrophotometry are used to compare and analyze the performance, and the experimental data of copper and cobalt are detected. The maximum relative error, average relative error, root mean square error of calibration (RMSEC), root mean square error of prediction (RMSEP) and correlation coefficient (R2) are used as evaluation indexes of the model, and the performance of different modeling methods is compared as follows.

**Figure 15:**
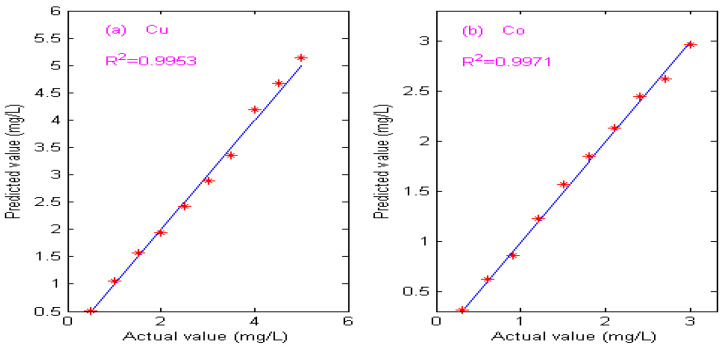
Calibration curve of predicted value and actual value. (**a**) Calibration curve of Cu. (**b**) Calibration curve of Co.

From Table 5, it can be seen that the detection performance of the proposed spectrophotometric method is much better than PLS and Kalman filtering methods. Due to the low sensitivity and narrow spectral effective band of copper and cobalt in high-concentration zinc solutions, the PLS and Kalman filtering methods have poor performance, and the average relative error is more than 10%, which cannot meet the industrial detection requirements. The prediction results of copper and cobalt concentration by the proposed spectrophotometric method are shown in Table 6, and the calibration curves of the predicted concentrations and the actual concentrations are shown in Figure 15.

As can be seen from Table 6, the RMSEP values of copper and cobalt are 0.098 and 0.063, respectively. The maximum relative error of copper is 5.20%, the average relative error is 3.77%, the maximum relative error of cobalt is 4.46%, and the average relative error is 2.85%. As can be seen from Figure 15, the predicted concentrations of copper and cobalt ions are very close to the actual concentrations, and the correlation coefficients (R^2^) are 0.9953 and 0.9971, respectively. The experimental results show that the detection accuracy of the proposed spectrophotometric method for copper and cobalt meets the requirements of actual production and is suitable for the simultaneous detection of copper and cobalt in high-concentration zinc solutions.

## 4. Experimental Method

### 4.1. Apparatus and Reagents

All chemicals were analytical reagents and needed no further purification. Deionized water was used for dissolution and dilution in the experiment. By using water as the solvent, 50 g/L zinc, 12.5 mg/L copper, 12.5 mg/L cobalt, and 12.5 mg/L nickel stock solutions were prepared. Then, standard solutions were prepared from the stock solution by continuous dilution as required. Sodium acetate buffer (pH = 5.5) was prepared by mixing the proper volumes of pure acetic acid and sodium acetate. Nitroso R salt solution (0.4%) was prepared as the chromogenic reagent, and hexadecyl trimethyl ammonium bromide (0.01 mol/L) was used as the stabilizer solution. A Beijing Puxi T9 Spectrometer was used to measure spectral data in the laboratory. The spectrophotometer was a high-sensitivity ultraviolet–visible spectrophotometer, equipped with a high-performance PMT receiver and special grating and mixed with a C-T monochromator and double beams; thus, it had high dynamic range and a good signal-to-noise ratio.

### 4.2. Procedures

In a 25 mL calibration flask, we added standard solutions of zinc, copper, cobalt, and nickel in different proportions, as well as 7.5 mL of acetic acid–sodium acetate buffer solution (pH = 5.5) and 5.00 mL of Nitroso R salt solution, and diluted them to the calibration scale with appropriate volumes of distilled water. A blank solution was prepared in the same way. The final concentration range of zinc was 20–30 g/L; for copper, 0.5–5.0 mg/L; for cobalt, 0.3–3.0 mg/L; and for nickel, 0.6–6.0 mg/L. A spectrometer was used to measure the spectral signal of the mixed solution of multi-metal ions against a high-zinc background, and it scanned at intervals of 1 nm in the wavelength range of 350 nm to 600 nm. All measured spectra are taken as the average of 5 repeated measurements. A total of 40 groups of mixed solutions in different proportions were prepared for spectral modeling. Among them, 30 groups of mixed solutions were used as the calibration set, and 10 groups were used as the prediction set.

## 5. Conclusions

In the purification process of zinc hydrometallurgy, the concentrations of copper and cobalt are much lower than that of matrix zinc, which leads to the serious masking of copper and cobalt signals by the matrix zinc signal and low sensitivity. Moreover, due to the similar chemical characteristics of polymetallic ions, the spectral signals overlap seriously. Aiming at the problems of low sensitivity, serious spectral overlap, and narrow effective band of copper and cobalt, a novel spectrophotometric method was developed to successfully detect the concentration of copper and cobalt in high-concentration zinc solutions. The proposed method uses the approximate derivative of the continuous wavelet transform to separate the spectral peaks and improve the resolution of copper and cobalt. Taking the information ratio and the separation degree as optimization indexes and the wavelet decomposition scale as a variable, a multi-objective optimization model is established, and the state transition algorithm is applied to solve the non-inferior solution to determine the optimal wavelet decomposition scale, thus improving the sensitivity and separation of copper and cobalt. By using derivative spectrometry combined with the zero-crossing technique, the calibration curves of copper and cobalt were established at fine zero-crossing points. The results show that the detection performance of the proposed method is far superior to the PLS and Kalman filtering methods, and the detection accuracy of copper and cobalt meets the requirements of actual production. The work proposed here is an interesting and promising attempt in the robust detection of trace copper and cobalt in high-concentration zinc solutions and may be applied to more fields.

## Figures and Tables

**Table 1:** Fitting accuracy of copper information ratio F_1_(x).

Wavelet Function	SSE	R-Square	Adjusted R-Square	RMSE
Haar	4.441 × 10^−5^	0.9941	0.9968	0.005332
Db4	3.036 × 10^−5^	0.9956	0.9981	0.002884
Coif3	0.0002908	0.9932	0.9944	0.007526
Sym3	0.0004756	0.9911	0.9916	0.009109

**Table 2:** Fitting accuracy of cobalt information ratio F_2_(x).

Wavelet Function	SSE	R-Square	Adjusted R-Square	RMSE
Haar	7.577 × 10^−5^	0.9974	0.9982	0.002352
Db4	0.0001518	0.9938	0.9949	0.006509
Coif3	0.0003512	0.9925	0.9936	0.007459
Sym3	0.0001308	0.9964	0.9967	0.004719

**Table 3:** Fitting accuracy of separation degree G(x).

Wavelet Function	SSE	R-Square	Adjusted R-Square	RMSE
Haar	8.754 × 10^−5^	0.9958	0.9973	0.002877
Db4	0.0003667	0.9922	0.9945	0.007905
Coif3	0.0001732	0.9943	0.9956	0.003562
Sym3	6.252 × 10^−5^	0.9971	0.9978	0.001304

**Table 4:** Statistical parameters of Cu and Co calibration curves under different wavelet bases.

Calibration Curve Parameters	CWT (Haar)		CWT (Db4)		CWT (Coif3)		CWT (Sym3)	
	Cu	Co	Cu	Co	Cu	Co	Cu	Co
Wavelength (nm)	529	573	554	531	516	542	513	561
Linear range (mg/L)	0.5−5	0.3−3	0.5−5	0.3−3	0.5−5	0.3−3	0.5−5	0.3−3
Slope	1.5518	2.9840	−1.3853	2.0076	1.9505	1.7413	−1.8463	−3.1337
Intercept	0.5330	0.0808	−0.2912	0.3055	0.2980	0.2803	−0.4760	−0.1229
Correlation coefficient (R^2^)	0.9952	0.9926	0.9896	0.9957	0.9915	0.9963	0.9931	0.9949
LOD (mg/L)	0.0829	0.0968	0.1087	0.0809	0.0991	0.0788	0.0862	0.0852
LOQ (mg/L)	0.2733	0.3198	0.3589	0.2642	0.3271	0.2575	0.2846	0.2814

**Table 5:** Performance comparison of different modeling methods.

Detection Ion	Evaluating Indicator	PLS	Kalman Filtering	The Proposed Method
Cu	Maximum relative error	18.57%	15.43%	5.20%
	Average relative error	13.35%	12.71%	3.77%
	RMSEC	0.894	0.629	0.154
	RMSEP	0.752	0.534	0.098
	Correlation coefficient	0.9886	0.9915	0.9953
Co	Maximum relative error	14.39%	13.58%	4.46%
	Average relative error	11.73%	10.62%	2.85%
	RMSEC	0.694	0.529	0.109
	RMSEP	0.427	0.376	0.063
	Correlation coefficient	0.9928	0.9942	0.9971

**Table 6:** Prediction results of simultaneous detection of copper and cobalt.

No.	Actual Value (mg/L)			Predicted Value (mg/L)		Relative Error (%)	
	Zn	Cu	Co	Cu	Co	Cu	Co
1	2.1 × 10^4^	0.50	0.60	0.513	0.619	2.60	3.17
2	2.2 × 10^4^	1.00	1.20	1.052	1.227	5.20	2.25
3	2.3 × 10^4^	1.50	1.80	1.571	1.846	4.73	2.56
4	2.4 × 10^4^	2.00	2.40	1.942	2.443	2.90	1.79
5	2.5 × 10^4^	2.50	3.00	2.419	2.959	3.24	1.36
6	2.6 × 10^4^	3.00	0.30	2.894	0.312	3.53	4.00
7	2.7 × 10^4^	3.50	0.90	3.357	0.861	4.09	4.33
8	2.8 × 10^4^	4.00	1.50	4.187	1.567	4.68	4.46
9	2.9 × 10^4^	4.50	2.10	4.677	2.133	3.94	1.57
10	3.0 × 10^4^	5.00	2.70	5.138	2.618	2.76	3.04
Average relative error (%)						3.77	2.85
RMSEP						0.098	0.063

## Data Availability

The data presented in this study are available upon request from the corresponding author.
